# Pain management after thoracotomy with dexamethasone and bupivacaine through a peripleural cather: a randomized controlled trial

**DOI:** 10.1186/s12871-024-02625-3

**Published:** 2024-07-16

**Authors:** Hamid Talebzadeh, Mohammad Eslamian, Erfan Sheikhbahaei, Ali Esparham, Hamidreza Zefreh, Pooria Sarblook, Alireza Firouzfar

**Affiliations:** 1 Department of Surgery, School of Medicine, Isfahan University of Medical Sciences, Isfahan, Iran; 2 Anesthesiology and Critical Care Research Center (ACCRC), Isfahan University of Medical Sciences, Isfahan, Iran; 3 Isfahan Minimally Invasive Surgery and Obesity Research Center, School of Medicine, Alzahra University Hospital, Isfahan University of Medical Sciences, Isfahan, Iran; 4 Student Research Committee, Faculty of Medicine, Mashhad University of Medical since, Mashhad, Iran; 5 Isfahan surgery department, Alzahra University Hospital, Sofe blvd, Isfahan, Iran

**Keywords:** Dexamethasone, Bupivacaine, Thoracotomy, Peripleural Catheter, Pain

## Abstract

**Introduction:**

Thoracotomy procedures can result in significant pain and cause nausea/vomiting. Glucocorticoids have anti-emetic and analgesic effects due to their anti-inflammatory and nerve-blocking properties. This study investigates the additive effect of local dexamethasone with bupivacaine as sole analgesic medication through a peripleural catheter after thoracotomy.

**Method:**

The study was conducted as a randomized control trial on 82 patients. Participants were allocated to receive either 2.5 mg/kg of bupivacaine plus 0.2 mg/kg of dexamethasone or 2.5 mg/kg of bupivacaine plus the same amount of normal saline as placebo through a 6 French peripleural catheter implemented above the parietal pleura and beneath the musculoskeletal structure of the chest wall. The primary outcome was the severity of pain 24 h after the operation in the visual analogue scale (VAS) score. Secondary outcomes were the incidence of nausea/vomiting, opioid consumption for pain control, and incidence of any adverse effects.

**Results:**

: A total of 50 participants were randomized to each group, and the baseline characteristics were similar between the groups. Median of VAS score (6 (3-8) vs. 8 (6-9), *p* < 0.001), postoperative opioid consumption (9 (36%) vs. 17 (68%) patients, *p*=0.024), and median length of hospital stay (4 (3-8) vs. 6 (3-12) days, *p* < 0.001) were significantly lower in the dexamethasone group. However, postoperative nausea/vomiting (*p*=0.26 for nausea and *p*=0.71 for vomiting) and surgical site infection (*p* = 0.55) were similar between the two groups.

**Conclusion:**

In thoracotomy patients, administering local dexamethasone + bupivacaine through a peripleural catheter can reduce postoperative pain, analgesic consumption, and length of hospital stay.

**Trial Registration:**

Iranian Registry of Clinical Trials (IRCT20220309054226N1, registration date: 3/21/2022.

## Introduction

Thoracotomy and its extensive incision on a part of the body with sensitive skin and rich sensory nerves brings significant pain after surgery, which may have negative consequences during the postoperative period if pain control is inadequate [1, 2]. These consequences may include complications such as atelectasis, pneumonia, and respiratory failure [1, 2]. In addition, chronic post-thoracotomy pain (CPTP) can be the result of poorly controlled acute pain following the surgery and persists for at least 2 months [3]. Therefore, perioperative analgesia is necessary to reduce postoperative pain (POP), prevent complications, and improve quality of life after thoracotomy.

Previous studies showed that glucocorticoids can be used as postoperative analgesic agents due to their nerve-blocking and anti-inflammatory properties in different types of surgeries [4–7]. In addition, a previous meta-analysis showed the beneficial role of intravenous dexamethasone in reducing POP and postoperative nausea/vomiting (PONV) [8]. However, the right dosage, efficacy, and the best route of administering it are not clear in thoracotomy. A critical analysis of enhanced recovery after thoracic surgery (ERATS) protocols underscored the ambiguity within the field. Among the five protocols scrutinized, each employed distinct techniques for POP management, ranging from oral medication to intravenous, intercostal, paravertebral, and epidural anesthesia [9]. Thoracic epidural analgesia (TEA) and thoracic paravertebral block (TPVB) are common approaches of POP management after thoracic surgery. Nevertheless, their implementation can be technically demanding and prone to considerable risks, such as pneumothorax, hematoma, dura puncture, infection, and nerve injury [10–13]. However, analgesia in peripleural area, where the injury has happened and is rich in somatic pain sensation, represents a regional, multilevel intercostal analgesic approach employed for managing POP. This technique involves the placement of a catheter above the parietal pleura at the end of the surgery under general anesthesia or direct thoracoscopic visualization. Importantly, no discernible impact on mobility, visceral physiologic functions, or blood pressure was found in previous investigations [14]. Peripleural analgesia is gaining popularity among alternate method of pain relief in thoracotomy, breast, and minimally invasive cardiothoracic surgery [15, 16]. Peripleural analgesia catheters enable a continuous administration of local anesthetic agents during the perioperative period to the exact intervention site. It is hypothesized that analgesia occurs by diffusion of local anesthetic into the parietal pleura, intercostal nerves, and intrathoracic sympathetic chain, thus providing adequate analgesia for unilateral thoracic and upper abdominal pain [17]. Previous studies have investigated the effect of subpleural multilevel intercostal continuous infusion of ropivacaine after thoracoscopic lung resection [14]. However, to date, no study has explored the effect of a bolus dose of dexamethasone via peripleural catheter on POP after thoracotomy. Therefore, the current study aims to investigate the effect of administering dexamethasone plus bupivacaine or bupivacaine plus placebo on POP, PONV, opioid consumption for pain control, and length of hospital stay by using a peripleural catheter before closing the chest wall in thoracotomy patients.

## Methods and materials

### Study design

This current randomized, double-blinded, placebo-controlled parallel trial was conducted between January 2022 and December 2022 at our university-affiliated hospital. All procedures performed in this study were in accordance with the ethical standards of the institutional and national research committee and the 1964 Helsinki declaration and its later amendments. Written informed consent was obtained from all of the participants before the enrollment. The protocol of this study was approved by the institutional review board of the Isfahan University of Medical Sciences and received approval number “IR.MUI.MED.REC.1400.811”. The protocol of this study has been submitted, registered, and approved by the Iranian Registry of Clinical Trials (IRCT20220309054226N1, registration date: 3/21/2022) and is available online through their website (irct.ir).

### Participants

The inclusion criteria comprised patients undergoing elective thoracotomy and lobectomy for conditions including lung hydatid cysts, bronchiectasis, and lung cancer, excluding cases with pleural metastasis. Participation in the study was contingent upon patient consent. Exclusion criteria were patients with a documented history of allergic reactions to glucocorticoids and/or bupivacaine, those who expired intraoperatively, lung cancer with pleural involvement, mesothelioma, asbestosis, opioid use disorder, and empyema. In addition, patients who were intubated more than 24 h after surgery were excluded from the study.

### Interventions

After posterolateral thoracotomy and before closing the pleura and chest wall, a 6 French, multi pore catheter was inserted between the intercostal muscles and the parietal pleura and then fixed to the skin. Participants were randomly assigned to either the dexamethasone + bupivacaine group (DPB) or the bupivacaine + placebo group. The combination of 2.5 mg/kg bupivacaine (5 mg/ml, Mylan Pharmaceutical Co. France) and 0.2 mg/kg dexamethasone (to a maximum dose of 14 mg, each ampule contains 4 mg/ml, Iran Hormone Pharmaceutical Company, Tehran, Iran) was administered via the catheter for the intervention group. The safety and efficacy of dexamethasone with dose of 0.2 mg/kg was shown previously [18]. The control group received 2.5 mg/kg of bupivacaine along with normal saline in an equivalent volume as given to the treatment group. The same dose in the same volume of bupivacaine was administered peripleurally via the catheter to both groups every 8 h till 24 h after the surgery. The chest tube was removed after 2–5 days with regard to full lung expansion in CXR imaging (no visible space between pleural layers).

### Randomization and blinding

The trial was conducted using the block randomization technique, in which with computer-generated randomizer software, two blocks each with 25 patients were used. This randomization was done by a trained analyst, without interference in the trial process and being blinded to the included patients and assessment of outcomes. The randomization process was concealed and sequentially numbered opaque sealed envelopes (SNOSE) were used. Both participants and involved investigators were blinded. A blinded pharmacist provided the medications from pharmaceutical companies and dexamethasone + bupivacaine or bupivacaine + placebo were prepared in identical syringes by the hospital pharmacy in operation due date and before finishing the surgery. To achieve this, the investigational and control medications were prepared and labeled by an independent research team not involved in direct patient care or outcome assessments. Each medication package was coded with a unique identifier that concealed its contents, preventing the pharmacist from discerning the treatment group. After terminating the trial and revealing the results, the groups of the study became unblinded.

### Outcome measures

Age, gender, medical history, weight, height, body mass index (BMI), hypertension, diabetes, and smoking were extracted from the patient’s medical records. The primary outcome was severity of the POP, and the secondary outcomes were frequency of PONV, postoperative opioid use on the next day after surgery for pain control, and incidence of any adverse events. The POP intensity was measured by the Visual Analogue Scale (VAS) with a 0–10 Likert scale 24 h after the operation. Any patient in pain received 10 mg/kg intravenous acetaminophen every six hours. In case of resistance to acetaminophen and severity of more than mild pain category on VAS [19, 20], a single shot of intramuscular pethidine (1 mg/kg) was administered. Morphine and intravascular administrations (either slow IV or infusion) were avoided due to the risk of apnea, atelectasis, and breath difficulties after thoracotomy and lung damages.

#### Sample size

According to Mao et al. study [21], considering alpha = 0.05, beta = 0.2, and what has been reported for the mean and standard deviations of VAS 24 h after thoracotomy (dexamethasone group: 0.81 ± 0.40, control group: 1.71 ± 1.08), a total of 28 patients was calculated to be sufficient for this study.

### Statistical analysis

Median (range) and frequency (%) were used for the presentation of numerical and categorical data, respectively. Chi-square test was used for comparison of categorical variables. Shapiro-Wilk test and Q-Q plot were used to assess the normal distribution of continuous data. Independent t-test and Mann-Whitney U test were used for normally and non-normally distributed continuous variables, respectively. A p-value less than 0.05 was considered statistically significant. All analyses were conducted using SPSS software version 26.0 (IBM Corp. USA).

## Results

### Participants

**Fig. 1:**
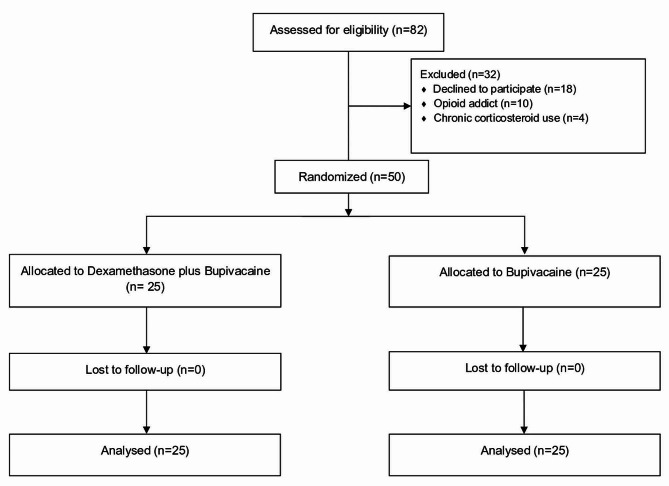
CONSORT flow diagram of study population

A total of 82 patients were screened for eligibility and 25 participants were randomized to each group. Figure 1 shows the CONSORT diagram of the present study. The baseline characteristics of the participants were similar between the two groups, with no significant differences in age, gender, BMI, hypertension, or smoking status. The duration of the operation was similar for both groups (Table 1).

**Table 1:** Demographic and pre-operative patients’ characteristics

Variable	Dexamethasone + Bupivacaine (*n* = 25)	Bupivacaine + placebo (*n* = 25)
**Age (years)**	36 (19–84)	36 (18–69)
**Male gender**,** n (%)**	18 (72%)	17 (68%)
**Weight (kg)**	73.2 ± 10.07	73.94 ± 13.57
**Height (cm)**	173.96 ± 9.09	171.92 ± 11.39
**BMI (kg/m** ^**2**^ **)**	24.24 ± 3.15	24.92 ± 2.97
**Hypertension**,** n (%)**	8 (32%)	6 (24%)
**Diabetes**,** n (%)**	7 (28%)	6 (24%)
**Smoking**,** n (%)**	15 (60%)	14 (56%)
**Operation duration (hours)**	2 (1.5-3)	2 (1.5–2.5)

All p-values are statistically nonsignificant

### Postoperative pain, analgesic consumption, and length of stay

**Fig. 2:**
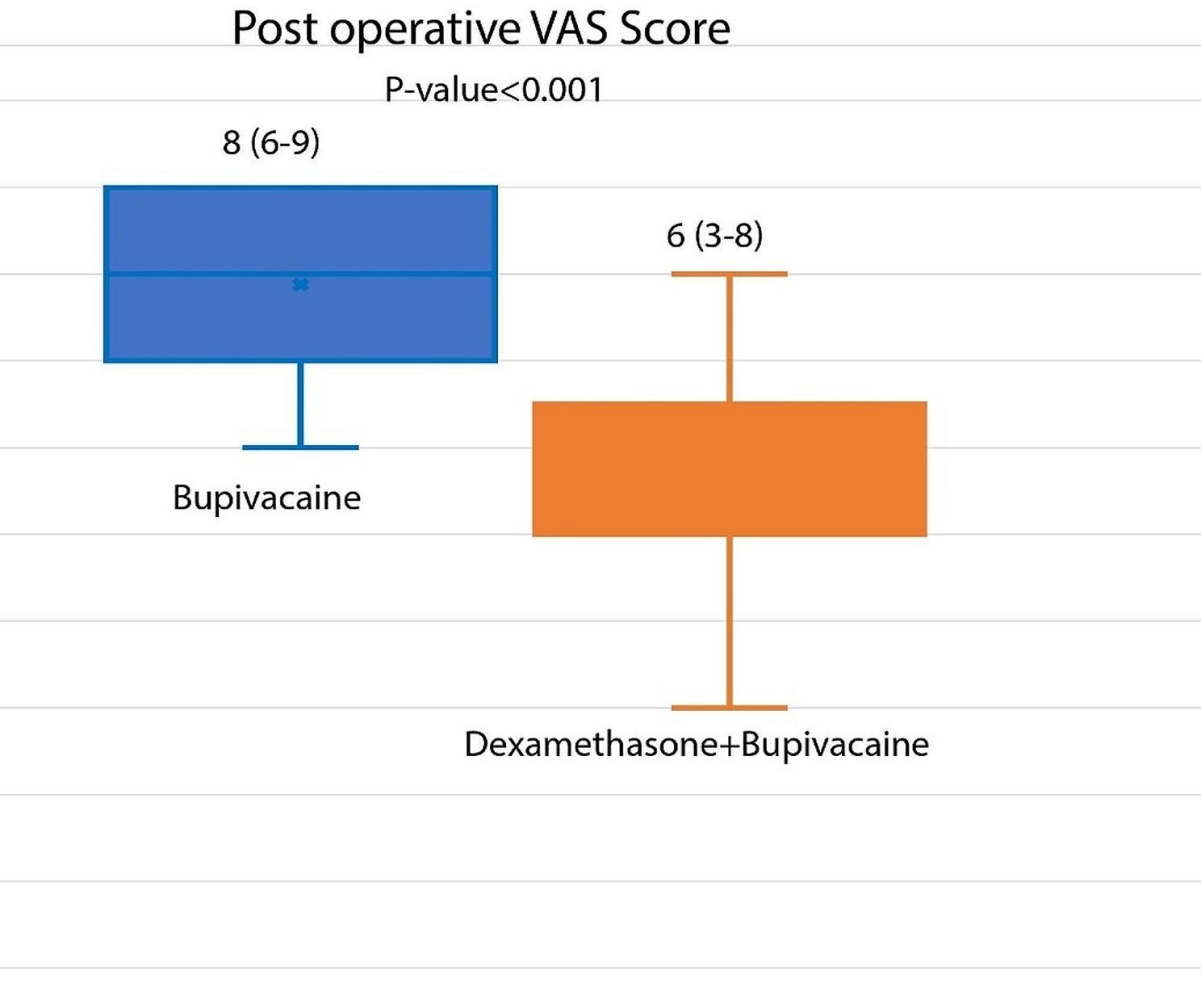
The visual analogue scale (VAS) score in dexamethasone + Bupivacaine group versus Bupivacaine + placebo group after thoracotomy

**Fig. 3:**
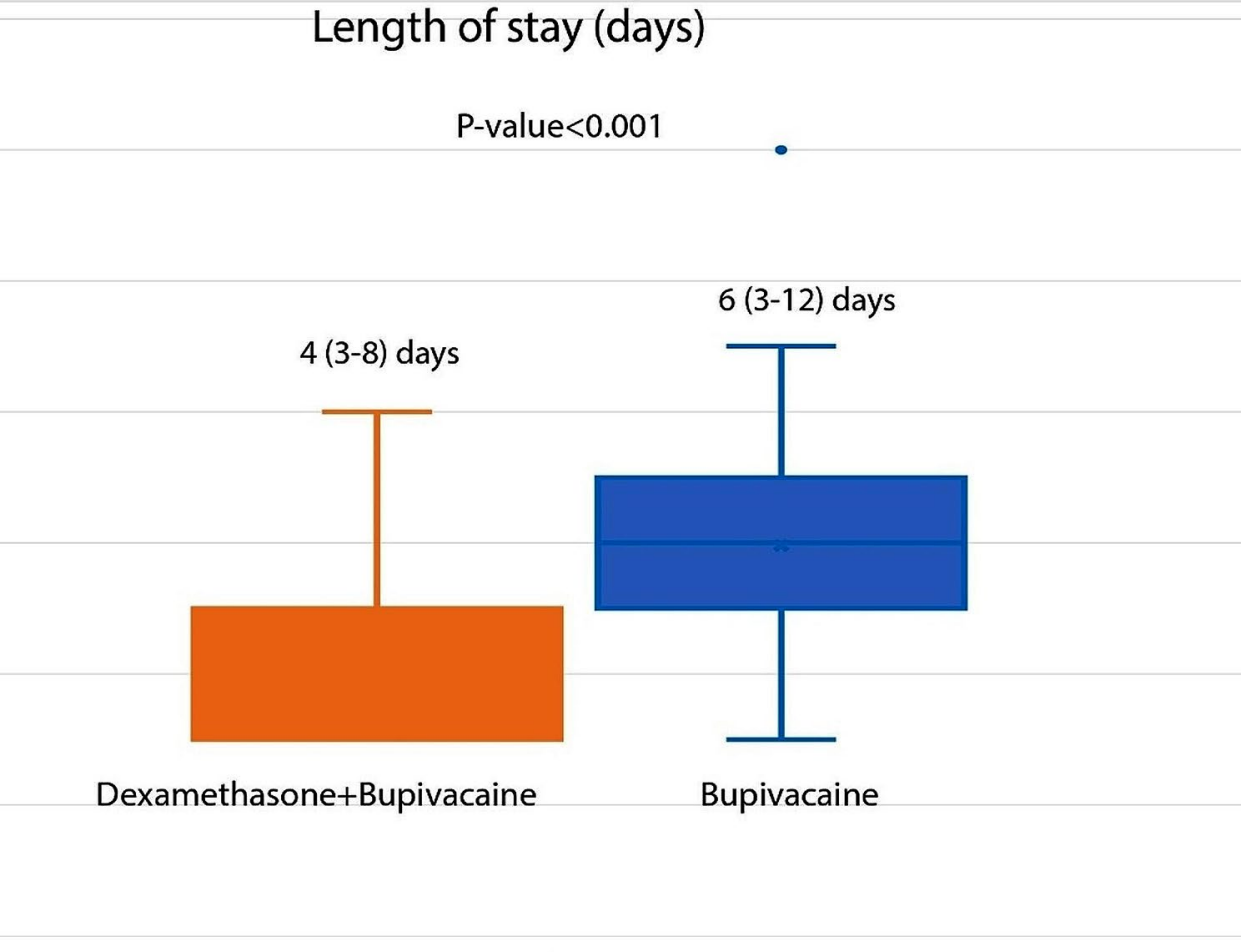
The hospital length of stays (days) in dexamethasone + Bupivacaine group versus Bupivacaine + placebo group after thoracotomy

The VAS score was significantly lower in the dexamethasone group (6 [3–8] vs. 8 [6–9], respectively, *p* < 0.001; Figure 2). The rate of opioid administration was significantly higher in the bupivacaine + placebo group (9 (36%) and 17 (68%) patients, respectively, *p* = 0.024). Length of hospital stay was significantly lower in the dexamethasone group (4 [3–8] and 6 [3–12] days, respectively, *p* < 0.001; Figure 3).

### Postoperative nausea, vomiting, and infection

Although PONV were lower in the dexamethasone group, the difference was not statistically significant (nausea: 10 (40%) vs. 14 (56%), respectively, *p* = 0.26 and vomiting: 4 (16%) vs. 5 (20%), respectively, *p* = 0.71). In addition, the rate of postoperative surgical site infection was not significantly different between the two groups (1 (4%) and 2 (8%) for dexamethasone + bupivacaine and placebo groups, respectively, *p* = 0.55).

## Discussion

To the best of our knowledge, this is the first study investigating the analgesic efficacy of a bolus dose of dexamethasone administered via peripleural catheter following thoracotomy. Our findings reveal that the combination of peripleural dexamethasone and bupivacaine yields an enhanced effect in mitigating postoperative pain (POP), reducing opioid usage, and shortening hospital stays in thoracotomy patients when compared to the administration of bupivacaine alone. However, the rate of PONV was not significantly different between groups. Therefore, we recommend anesthesiologists, general, thoracic, and trauma surgeons to test peripleural catheter for their patients.

Thoracotomy is commonly regarded as one of the most excruciating surgical procedures, and the resulting POP is frequently intense and challenging to treat [22]. POP following thoracic surgery poses a significant clinical challenge, often linked to heightened morbidity and mortality rates. Research indicates that inadequate pain control can result in severe pulmonary complications, including impaired secretion clearance, mucous plugging, and atelectasis [23]. Previous studies used different approaches to administer dexamethasone to thoracotomy patients such as intravenous; however, neither of them evaluated peripleural way. In this study, we employed a 6 F peripleural catheters, being placed beneath the musculoskeletal structure and above the parietal pleura to administer local anesthetics into numerous intercostal levels, where the exact intervention is and highest pain sensation is coming from. It has been proposed that local anesthesia solution diffuses out of the pleural cavity, blocking multiple intercostal nerves, the sympathetic chain of the upper extremity, the brachial plexus, splanchnic nerves, phrenic nerves, coeliac plexus, and ganglia, producing effective analgesia both above and below the diaphragm, even though the exact mechanism of action of intra-pleural analgesia is unclear [24]. The most plausible pathway for fluid transmission in the parietal pleura is the stomata that are located between the mesothelial cells. Studies on cadavers showed that Indian ink spread from the parietal pleura into the peripleural area and returned to several intercostal nerves. This served as the foundation for our study’s positioning methodology [25–27].

Prior research reported inconsistent findings when comparing the impact of peripleural analgesia with alternative methods for reducing POP. Jung et al. conducted a study comparing peripleural analgesia with intravenous patient-controlled analgesia and reported similar average pain scores between the two methods [28]. Furthermore, in 2011, Hotta et al. conducted a randomized study comparing TEA to extrapleural continuous analgesia for patients with video-assisted thoracoscopic surgery. The requirement for rescue analgesia and pain levels on the visual analog scale did not differ significantly, according to their findings [29]. On the contrary, Tezcan et al. showed that in the peripleural analgesia group, all patients necessitated rescue analgesia, whereas in the TEA group, five patients (33%) required rescue analgesia. Additionally, patients who received peripleural analgesia demonstrated higher visual analogue scores both at rest and during coughing compared to those who received TEA [30]. Furthermore, Kanazi et al. demonstrated that patients who underwent peripleural analgesia experienced higher visual analogue scores both at rest and during coughing compared to those who received TEA [31]. These conflicting results may stem from variations in analgesic types, dosages, patient comorbidities, and the diverse types of thoracotomies involved.

However, when using peripleural analgesia, several advantages should be considered. Patients with properly positioned TEA are frequently labeled as “immobilized” even if their legs continue to have motor function. However, patients with peripleural analgesia do not have a loss of motor function [14]. In addition, significant post-void residuals were observed in individuals with TEA in a prospective trial; however peripleural analgesia does not present this issue [32]. Another advantage of peripleural analgesia in comparison to TEA and other regional analgesic techniques, such as paravertebral block, intercostal block, and erector spinae block, is its rapid placement under direct vision and the absence of additional equipment or specialized staff (except for the catheter). Furthermore, patients benefit from the placement of the analgesic catheter under general anesthesia, as opposed to the potentially stressful awake placement required for a thoracic epidural catheter or awake percutaneous regional analgesic techniques [14, 33].

Our results should be interpreted by considering several limitations. First, the long-term results of dexamethasone and its effect on CPTP were not investigated. Second, the VAS score was only assessed after 24 h, potentially overlooking variations in pain levels during the immediate postoperative period and later recovery stages. Future research should include multiple assessments of various subjective and objective questionnaires evaluating different aspects of postoperative quality at various intervals post-thoracotomy, such as immediately after surgery, at several points during the first 24 h, and over the subsequent recovery period. Third, we were unable to perform a dose-response analysis between dexamethasone and VAS. Further research is warranted to determine the optimal dosing and timing of dexamethasone in this setting.

## Conclusion

Local dexamethasone administration through a peripleural catheter can significantly reduce postoperative pain, opioid consumption, and length of hospital stay after thoracotomy. These findings highlight the potential benefits of utilizing dexamethasone as an effective and safe additive to current postoperative pain management strategies, such as bupivacaine. However, it did not show an anti-emetic effect in this group.

## Data Availability

The datasets used and/or analysed during the current study available from the corresponding author on reasonable request.
